# Contribution of GIP and GLP-1 to the Insulin Response to Oral Administration of Glucose in Female Mice

**DOI:** 10.3390/biomedicines11020591

**Published:** 2023-02-16

**Authors:** Bo Ahrén

**Affiliations:** Department of Clinical Sciences Lund, Lund University, Sölvegatan 19, 22185 Lund, Sweden; bo.ahren@med.lu.se

**Keywords:** GIP, GLP-1, incretin, mice

## Abstract

It has previously been shown that the incretin effect accounts for ≈50% of the insulin response to oral glucose in normal mice. Now, I have proceeded and studied the contribution of glucose-dependent insulinotropic polypeptide (GIP) and glucagon-like peptide-1 (GLP-1) to the insulin response to oral glucose in female mice by using receptor antagonists. A specific GIP receptor antagonist (mGIP(3-30); 50 or 500 nmol/kg), a specific GLP-1 receptor antagonist (exendin(9-39); 3 or 30 nmol/kg), the combination of mGIP (500 nmol/kg) and exendin(9-39) (30 nmol/kg), or saline was given intravenously four minutes after administration of glucose (50 mg) through a gastric tube in anesthetized C57/BL6J mice (*n* = 95) with samples obtained before glucose administration and after 15, 30 and 60 min. The insulinogenic index, determined as the area under the 60 min curve for insulin (AUC_insulin_) divided by the AUC_glucose_, was used to reflect the insulin response. It was found that the insulinogenic index was reduced by 67 ± 4% by mGIP(3-30) (*p* < 0.001), by 60 ± 14% by exendin(9-39) (*p* = 0.007) and by 61 ± 14% by the combination of mGIP(3-30) and exendin(9-39) (*p* = 0.043), both at their highest doses, compared to animals injected with glucose in the same experimental series. It is concluded that both GIP and GLP-1 are required for a normal incretin effect in female mice, that they contribute similarly to the insulin response, and that it is unlikely that there is another incretin hormone in this species.

## 1. Introduction

As demonstrated in 1964 in human studies, the insulin response is markedly augmented after oral glucose compared to intravenous glucose at identical glucose levels [1,2]. This is the basis for the incretin effect which is an important physiological process to assure a sufficient insulin response not only after oral glucose but also after meal ingestion, as reviewed several decades ago [3]. The incretin effect is responsible for ≈40–65% of the insulin response to oral glucose in humans [4,5]. It is mainly mediated by the two gut incretin hormones, glucose-dependent insulinotropic polypeptide (GIP) and glucagon-like peptide-1 (GLP-1); these hormones are both released after the ingestion of oral glucose or meal and potentiate glucose-stimulated insulin secretion at dose levels achieved after meal ingestion [6,7,8,9,10].

The relative contribution of the two incretin hormones to the incretin effect has been examined using different strategies. A study in healthy humans used the specific GIP receptor antagonist GIP(3-30) and the specific GLP-1 receptor antagonist exendin(9-39) [10]. It was found that the insulin response to oral glucose was reduced more by the GIP antagonist than by the GLP-1 antagonist, suggesting that GIP is more important than GLP-1. This was also concluded in a recent review [6]. Another study on healthy humans used infusions of physiological levels of the two hormones during stepwise glucose clamp and meal ingestion [11]. This study showed that the two hormones nearly equally contribute to the incretin effect.

Since model experiments in mice are important in biomedical research, it is of relevance to examine the incretin effect in this species also. It has thus been demonstrated that the incretin effect indeed exists in mice. This was demonstrated in a study in which glucose was infused intravenously at a variable rate to match glucose levels achieved after the oral administration of 25 mg glucose [12]. The results showed that the insulin response was double after the oral administration of glucose compared to the intravenous infusion [12]. This would suggest that the incretin effect accounts for ≈50% of the insulin response to oral glucose in mice. It is also known that GIP and GLP-1 levels increase after oral glucose in mice [8,13]. Furthermore, both GIP and GLP-1 have robust effects to stimulate insulin secretion in mice when administered intravenously in mice [14]. Moreover, in mice with double deletion of the GIP and GLP-1 receptors, the insulin response to oral glucose is reduced by ≈65% [15]. It is thus clear that GIP and GLP-1 are important incretin hormones in mice also.

However, the contribution of each of the incretin hormones for the incretin effect in mice is not known. In this study, I have used the approach of administering GIP or GLP-1 receptor antagonists, alone or together, to study the relative contribution of GIP and GLP-1 to the insulin response to oral glucose in female mice. I used exendin(9-39), which has been used in previous studies in my laboratory in mice [16,17]. As GIP receptor antagonist, I used the recently established antagonist GIP(3-30), which in its human form has been successful in inhibiting the actions of GIP [18], and which in its murine form (mGIP(3-30)), has been characterized in mice [19,20]. These antagonists were administered alone or together and in association with gastric glucose administration. The glucose and insulin responses were determined. Since the glucose levels after glucose administration were altered by the receptor antagonists and therefore not matched between experimental groups and controls, the indirect measure of insulinogenic index was used to estimate the beta cell response.

## 2. Methods

### 2.1. Animals

Female C57BL/6J mice from Taconic, Skensved, Denmark were used. They were 4–6 months of age, maintained in a temperature-controlled room (22 °C) on a 12:12 h light-dark cycle (light on at 7:00 A.M.) and fed a standard pellet diet (energy 14.1 MJ/kg with 14% from fat, 60% from carbohydrate and 26% from protein; SAFE, Augy, France) and tap water ad libitum. Only female mice were used, to avoid the stress of single housing, which is used in male mice.

### 2.2. Experiments

A total of 95 animals were allocated for the study (mean body weight (±SEM) was 20.6 ± 0.2 g). Food was removed from the cages at 7:30 A.M. At 9:30 A.M., anaesthesia was induced with Fluafent (i.e., a mixture of fluanisone and fentanyl citrate) and midazolam, as previously described [21]. In short, 10 mg fluanisone (Key Organics, Camelford, Cornwall, UK) was dissolved in 1 mL sterile water at 70 °C for 60 min. This solution was mixed with 1 mL of fentanyl citrate (Sigma-Aldrich, St. Louis, MO, USA; 0.315 mg/mL); 100 µL of this solution were given intraperitoneally to each mouse (0.016 mg fentanyl citrate and 0.5 mg fluanisone/mouse). Midazolam (0.167 mg/mouse; Roche, Basel, Switzerland) was also given (100 µL/mouse). Fifteen minutes later, 50 mg of glucose (Sigma-Aldrich, St. Louis, MO, USA, dissolved in saline) were administered through a gastric tube (outer diameter 1.2 mm). Four minutes later, mice were injected intravenously in a tail vein (10 µL/g body weight given over 3 s) with synthetic exendin(9-39) (Sigma-Aldrich, 3 or 30 nmol/kg, dissolved in saline), synthetic mGIP(3-30) (CASLO ApS, Kongens Lyngby, Denmark; 50 or 500 nmol/kg; dissolved in saline with addition of NaOH; final concentration 60 µmol/L), the combination of exendin(9-39) (30 nmol/kg) and mGIP(3-30) (500 nmol/kg), or saline. Whole blood was sampled in heparinized pipettes from the intraorbital retrobulbar sinus plexus (40 µL) at 0, 15, 30 and 60 min. Plasma was separated by centrifugation and stored at −20 °C until analysis. The experiments were undertaken in batches of 6–8 mice on each experimental day by one experienced technician. Animals given glucose alone were always included in the experimental series with receptor antagonists.

### 2.3. Analyses

Glucose in whole blood was detected with the glucose oxidase method using AccuChek Aviva (Hoffman-La Roche, Basel, Switzerland). Insulin was determined by ELISA (Mercodia, Uppsala, Sweden). The intra-assay coefficient of variation (CV) of the method is 4% at low and high levels, and the inter-assay CV is 5% at low and high levels. The lower limit of the assay is 6 pmol/L.

### 2.4. Statistics

All individual results from the completer population were included in the analysis. Data are presented as means ± SEM. Suprabasal (incremental) areas under the curves (AUCs) were calculated with the trapezoid rule. The insulinogenic index was estimated by dividing AUC_insulin_ by AUC_glucose_. Whether AUC_glucose_, AUC_insulin_ and the insulinogenic index were normally distributed was tested by analyzing the estimates after glucose administration alone in all animals, using the Kolmogorov–Smirnov test. Since the data points for AUC_glucose_ and AUC_insulin_ were normally distributed and the data points for the insulinogenic index were not normally distributed, differences between groups were determined using Student’s *t* test for tests of significance for AUC_glucose_ and AUC_insulin_ and non-parametric tests were used for tests of significance for insulinogenic index. Tests were performed between animas tested in the same experimental series. For example, groups of animals given mGIP(3-30) at 50 nmol/kg had their glucose controls and animals given mGIP(3-30) at 500 nmol/kg had their glucose controls. Statistical tests were performed by SPSS, v 28 (IBM Corp., Statistics for Windows, Version 28.0. Armonk, NY, USA). Statistical significance was defined as *p* < 0.05.

## 3. Results

### 3.1. Glucose Controls

Supplementary Figure S1 shows glucose and insulin data in all animals given glucose alone (*n* = 45). It is seen that glucose levels peaked at 30 min and insulin levels peaked at 15 min. The figure also shows individual data points for AUC_glucose_, AUC_insulin_ and the insulinogenic index. The Kolmogorov–Smirnov test for normality showed that the individual data points for AUC_glucose_ (*p* = 0.18) and AUC_insulin_ (*p* = 0.20) were normally distributed, whereas the data points for the insulinogenic index were not normally distributed (*p* = 0.001).

### 3.2. GIP Receptor Antagonism

**Figure 1:**
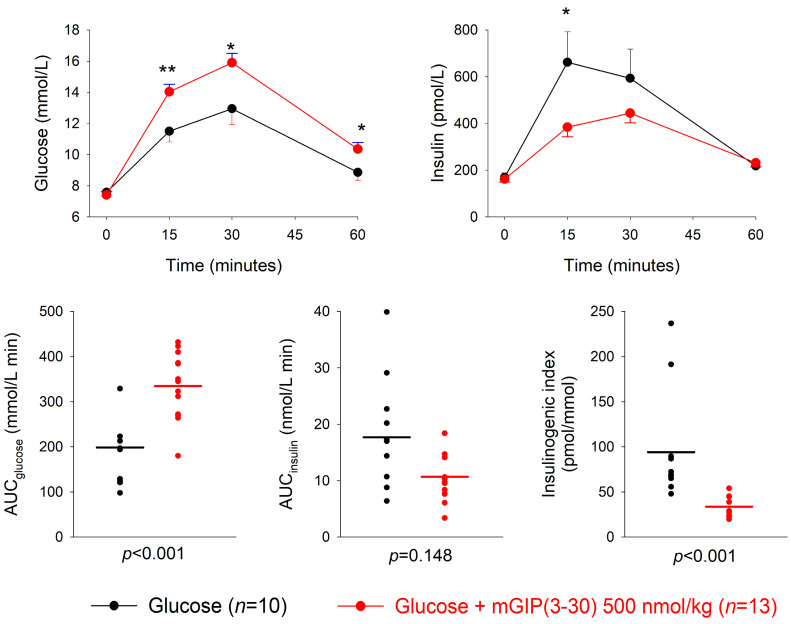
Glucose and insulin levels and individual data points for AUC_glucose_, AUC_insulin_ and insulinogenic index after intravenous administration of the GIP receptor antagonist mGIP(3-30) (500 nmol/kg) or saline four minutes after an oral administration of 50 mg glucose in normal mice. Means ± SEM are shown. Asterisks indicate the probability level of random difference between mGIP(3-30) versus the glucose control; * *p* < 0.05; ** *p* < 0.01; exact *p* levels see results section; *n* indicates number of animals.

The GIP receptor antagonist mGIP(3-30) or saline was given intravenously at four minutes after the oral administration of 50 mg glucose. At the low dose of 50 nmol/kg, mGIP(3-30) did not significantly affect the glucose or insulin responses to glucose, except a slightly higher glucose level in the mGIP(3-30) group at 30 min (*p* =0.031) (Supplementary Figure S2). At 500 nmol/kg, mGIP(3-30) significantly increased the 15, 30 and 60 min glucose levels (*p* = 0.008, *p* = 0.014 and *p* = 0.026, respectively) and significantly reduced the 15 min insulin levels (*p* = 0.010) compared to glucose alone (Figure 1). Furthermore, AUC_glucose_, AUC_insulin_ and the insulinogenic index were not significantly different between mGIP(3-30) at 50 nmol/kg and the glucose control (Table 1). In contrast, AUC_glucose_ was significantly higher after mGIP(3-30) at 500 nmol/kg compared to glucose alone (*p* < 0.001), whereas AUC_insulin_ did not differ significantly between the groups (*p* = 0.148). The insulinogenic index was significantly lower in the mGIP(3-30) group (*p* < 0.001) (Table 1). The mean insulinogenic index was reduced by 67 ± 4% (*p* < 0.001) by mGIP(3-30) at 500 nmol/kg compared to glucose alone. 

### 3.3. GLP-1 Receptor Antagonism

**Figure 2:**
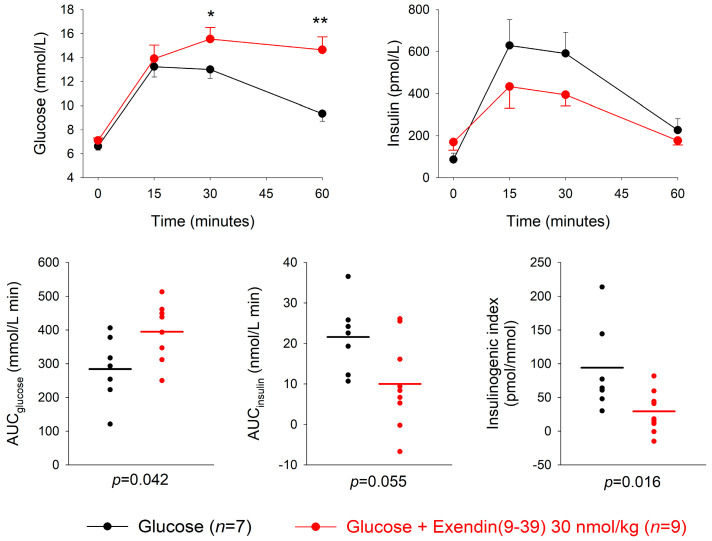
Glucose and insulin levels, and individual data points for AUC_glucose_, AUC_insulin_ and insulinogenic index after intravenous administration of the GLP-1 receptor antagonist exendin(9-39) (30 nmol/kg) or saline four minutes after oral administration of 50 mg glucose in normal mice. Means ± SEM are shown. Asterisks indicate the probability level of random difference between exendin(9-39) versus glucose control; * *p* < 0.05; ** *p* < 0.01; exact *p* levels see results section; *n* indicates number of animals.

The GLP-1 receptor antagonist exendin(3-39) or saline was given at four minutes after the oral administration of 50 mg glucose. At the low dose of 3 nmol/kg, exendin(9-39) did not significantly affect the glucose or insulin responses to glucose at any time point (Supplementary Figure S3). In contrast, at the high dose of 30 nmol/kg, exendin(9-39) significantly increased the 30 and 60 min glucose levels (*p* = 0.025 and *p* = 0.002, respectively) with a reduction in insulin levels compared to glucose alone, which however, did not reach significance in any individual time point (Figure 2). Furthermore, AUC_glucose_, AUC_insulin_ and insulinogenic index were not significantly different between exendin(9-39) at 3 nmol/kg and the glucose control (Table 1). In contrast, AUC_glucose_ was significantly higher after exendin(9-39) at 30 nmol compared to glucose alone (*p* = 0.042), whereas AUC_insulin_ was not significantly different between the groups (*p* = 0.055). The insulinogenic index was significantly lower in the exendin(9-39) group (*p* = 0.016) (Table 1). The mean insulinogenic index was reduced by 60 ± 14% (*p* = 0.007) by exendin(9-39) at 30 nmol/kg compared to glucose alone.

### 3.4. Combination of GIP and GLP-1 Receptor Antagonism

**Figure 3:**
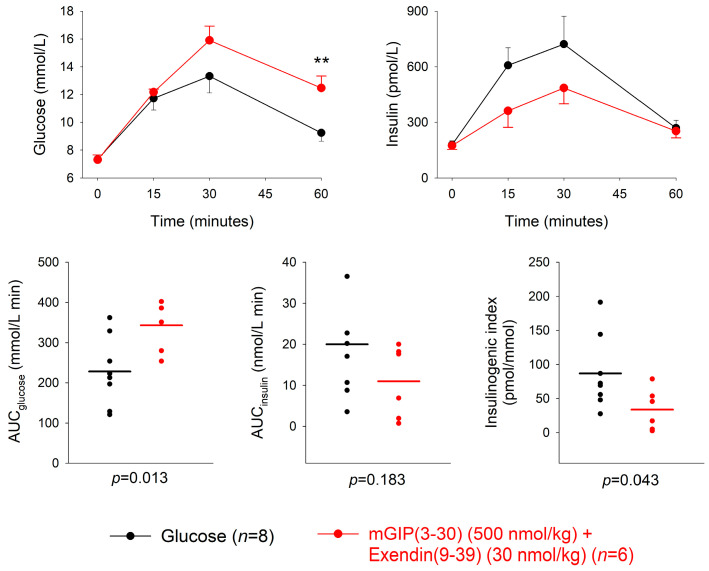
Glucose and insulin levels, and individual data points for AUC_glucose_, AUC_insulin_ and insulinogenic index after combined intravenous administration of the GIP receptor antagonist mGIP(3-30) (500 nmol/kg) and the GLP-1 receptor antagonist exendin(9-39) (30 nmol/kg) or saline four minutes after oral administration of 50 mg glucose in normal mice. Means ± SEM are shown. Asterisks indicate the probability level of random difference between mGIP(3-30) + exendin(9-39) versus the glucose control; ** *p* < 0.01; exact *p* levels see results section; *n* indicates number of animals.

The combination of GIP receptor antagonist mGIP(3-30) (500 nmol/kg) and the GLP-1 receptor antagonist exendin(9-39) (30 nmol/kg) or saline was given intravenously at four minutes after the oral administration of 50 mg glucose. The combination significantly increased the 60 min glucose levels (*p* = 0.008) and reduced the insulin levels, although the difference in insulin levels between combined treatment and glucose alone did not reach significance at any individual time point (Figure 3). Furthermore, AUC_glucose_ was significantly higher after mGIP(3-30) plus exendin(9-39) compared to glucose alone (*p* = 0.013), whereas AUC_insulin_ was not significantly different (*p* = 0.183). The insulinogenic index was significantly lower in the combination group compared to glucose alone (*p* = 0.043) (Table 1). The mean insulinogenic index was reduced by 61 ± 14% (*p* = 0.043) by the combination of GIP and GLP-1 receptor antagonism with glucose compared to glucose alone.

## 4. Discussion

In this study, the specific GIP receptor antagonist mGIP(3-30) and the specific GLP-1 receptor antagonist exendin(9-39) were used to examine the contribution of GIP and GLP-1 in the insulin response to gastric glucose administration in mice. Exendin(9-39) is a well established GLP-1 receptor antagonist, the use of which was recently reviewed [22]. Exendin(9-39) has been verified to be a GLP-1 receptor antagonist in several mouse studies [16,17]. Exendin(9-39) has also been used in several human studies to inhibit GLP-1 activity [5,10,23,24]. GIP(3-30) is, in contrast, a novel antagonist. It is a naturally occurring GIP fragment, which has been shown to inhibit GIP receptors [18,25] and to be efficient to inhibit GIP receptors also in mice [19,20]. In one study, however, mGIP(3-30) did not affect the glucose and insulin response to oral glucose in mice [20]. In that study, the antagonist was administered subcutaneously 15 min before glucose, which is probably too early due to the short half-life of the antagonist. In contrast, in the present study on mGIP(3-30) was administered at four minutes after oral glucose intravenously, which may have preserved an effect.

The present results show that the insulinogenic index after the oral administration of glucose in normal mice is reduced by 60–70% by mGIP(3-30) and exendin(9-39), both when the antagonists were given alone and when they were given in combination. These effects were associated with increased glucose levels. This shows that the two incretin hormones GIP and GLP-1 equally reduce the insulin response to oral glucose in mice. The result also shows that each of GIP and GLP-1 largely contributes to the incretin effect in mice. Previously, it was demonstrated that the overall incretin effect contributes to the insulin response to oral glucose by ≈50% (the remaining dependent on the increased glucose levels) in mice [12]. This also confirms a previous study in mice with double deletion of GIP and GLP-1 receptors, where the insulin response was reduced by ≈65% [15]. A similar magnitude of contribution by the incretin effect on the insulin response to oral glucose has been demonstrated in humans [4,5,26,27]. 

Previous studies have used individual incretin receptor knockout mice to analyze the contribution of GIP and GLP-1 for the incretin effect. Most of these studies have shown that the insulin response to oral glucose is reduced by ≈30–50% in these mice [28,29,30,31,32,33], although no effect has also been found [30,33,34]. The lower reduction in insulin response in knockout mice than in the present study using receptor antagonists may be explained by compensatory mechanisms in the receptor knockout mice. Thus, in GIP receptor knockout mice, there is an adaptive upregulation of beta cell function which may counterbalance a reduced action of GIP [16]. Similarly, in GLP-1 receptor knockout mice, there is an upregulation of the GIP response to oral glucose, similarly potentially counterbalancing a reduced action of GLP-1 [17]. Single receptor knockout mice may therefore not be optimal for the study on the relative contribution of GIP versus GLP-1.

A limitation of the study is that it can not be excluded that higher doses of the antagonists would have had even more pronounced inhibitory actions. However, for exendin(9-39), the high dose used (30 nmol/kg) has previously been shown to abolish the GLP-1 induced insulin secretion in mice [16,17]. Furthermore, for mGIP(3-30), a dose of 50 nmol/kg was suggested to result in a complete inhibition in previous studies [19] and in the present study, a dose of more than 10 times higher was used. Therefore, it is unlikely that the doses were too low. Other potential limitations of the study are that anesthetized mice were used, which on one hand relieves the stress seen in studies with unanesthetized animals but on the other hand may have allowed other effects. I also used only female mice, and therefore the current conclusions are valid for female mice. Finally, the insulinogenic index was used to reflect the insulin response to oral glucose. This was considered a more appropriate estimate than, for example, insulin levels or AUC_insulin_, since the glucose levels by study design were not matched in the different experimental groups.

In summary, by using specific GIP and GLP-1 receptor antagonists, this study showed that the insulin response to oral glucose is reduced by more than 60% after each of them and in combination in female mice. This shows that both GIP and GLP-1 are required for a normal incretin effect, that they contribute similarly to the insulin response, and that it is unlikely that there is another incretin hormone in this species.

## Figures and Tables

**Table 1:** AUC_glucose_, AUC_insulin_ and insulinogenic index after intravenous administration of the GIP receptor antagonist mGIP(3-30) (50 or 500 nmol/kg), the GLP-1 receptor antagonist exendin(9-39) (3 or 30 nmol/kg), the combination of mGIP(3-30) (500 nmol/kg) and exendin(9-39) (30 nmol/kg), or saline (= glucose alone) four minutes after oral administration of 50 mg glucose in normal mice. Means ± SEM are shown. *n* indicates number of animals in each group; *p* indicates the probability level of random difference between the experimental groups.

	Dose of Antagonist (nmol/kg)	*n*	AUC_glucose_ mmol/L min	*p*	AUC_insulin_ nmol/L min	*p*	Insulinogenic Index (pmol/ mmol)	*p*
mGIP(3-30) + glucose	50	14	258.5 ± 15.5	0.45	17.4 ± 1.7	0.32	71.5 ± 8.6	0.076
Glucose alone		12	233.1 ± 30.9		20.1 ± 1.9		107.2 ± 16.1	
mGIP(3-30) + glucose	500	13	334.7 ± 20.6	<0.001	10.7 ± 1.1	0.148	33.8 ± 4.3	<0.001
Glucose alone		10	198.8 ± 28.1		17.7 ± 3.6		94.2 ± 21.0	
Exendin(9-39) + glucose	3	8	272.3 ± 33.9	0.80	18.0 ± 3.4	0.44	71.1 ± 12.0	0.44
Glucose alone		8	260.5 ± 30.6		14.8 ± 3.9		53.8 ± 12.8	
Exendin(9-39) + glucose	30	9	394.6 ± 32.8	0.042	10.0 ± 3.6	0.055	29.4 ± 10.2	0.016
Glucose		7	284.3 ± 36.6		21.6 ± 3.3		91.1 ± 24.6	
mGIP(3-30) + Exendin(9-39) + glucose	500 (mGIP) 30 (exendin)	6	343.1 ± 25.2	0.013	11.0 ± 3.6	0.183	33.8 ± 12.4	0.043
Glucose alone		8	228.4 ± 30.2		20.0 ± 4.6		86.9 ± 19.2	

## Data Availability

Original data are available upon reasonable request to the author.

## Supplementary Materials

The following supporting information can be downloaded at: https://www.mdpi.com/article/10.3390/biomedicines11020591/s1, Figure S1: Glucose and insulin levels, and individual data points for AUCglucose, AUCinsulin and insulinogenic index after an oral administration of 50 mg glucose in normal mice (*n* = 45); Figure S2: Glucose and insulin levels and individual data points for AUCglucose, AUCinsulin and insulinogenic index after intravenous administration of the GIP receptor antagonist mGIP(3-30) (50 nmol/kg) or saline four minutes after an oral administration of 50 mg glucose in normal mice; Figure S3: Glucose and insulin levels, and individual data points for AUCglucose, AUCinsulin and insulinogenic index after intravenous administration of the GLP-1 receptor antagonist exendin(9-39) (3 nmol/kg) or saline four minutes after oral administration of 50 mg glucose in normal mice.
