# Supplementary material for: Contribution of GIP and GLP-1 to the Insulin Response to Oral Administration of Glucose in Female Mice
Source: Biomedicines. 2023 Feb 16;11(2):591. doi: 10.3390/biomedicines11020591 (PMC9953110; doi:10.3390/biomedicines11020591)
Supplement: Supplementary file 1 [file biomedicines-11-00591-s001.zip › biomedicines-2201449-supplementary.pdf]

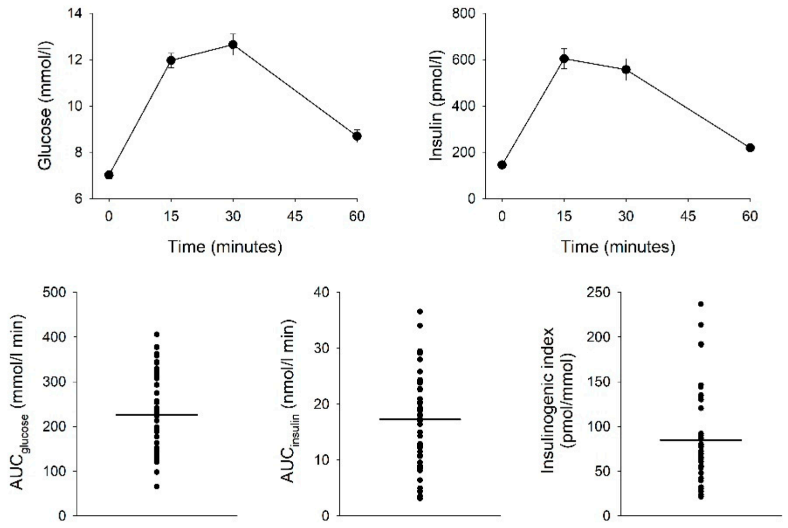

Supplementary Figure S1. Glucose and insulin levels, and individual data points for $AUC_{glucose}$, $AUC_{insulin}$ and insulinogenic index after an oral administration of 50mg glucose in normal mice (n=45). Means±SEM are shown.

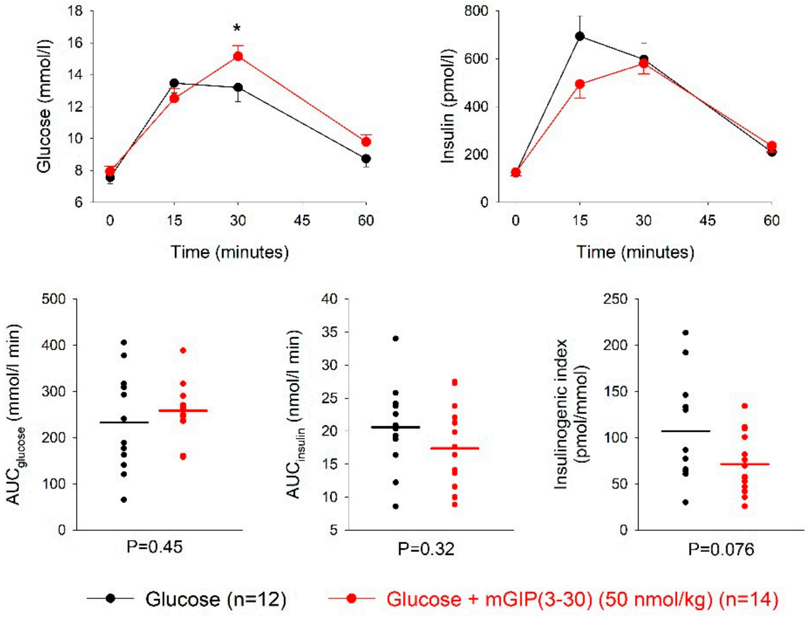

Supplementary Figure S2. Glucose and insulin levels and individual data points for $AUC_{glucose}$, $AUC_{insulin}$ and insulinogenic index after intravenous administration of the GIP receptor antagonist mGIP(3-30) (50 nmol/kg) or saline four minutes after an oral administration of 50mg glucose in normal mice. Means±SEM are shown. Asterisks indicate probability level of random difference between mGIP(3-30) versus glucose control; *P<0.05; exact P levels see result section. n indicates number of animals.

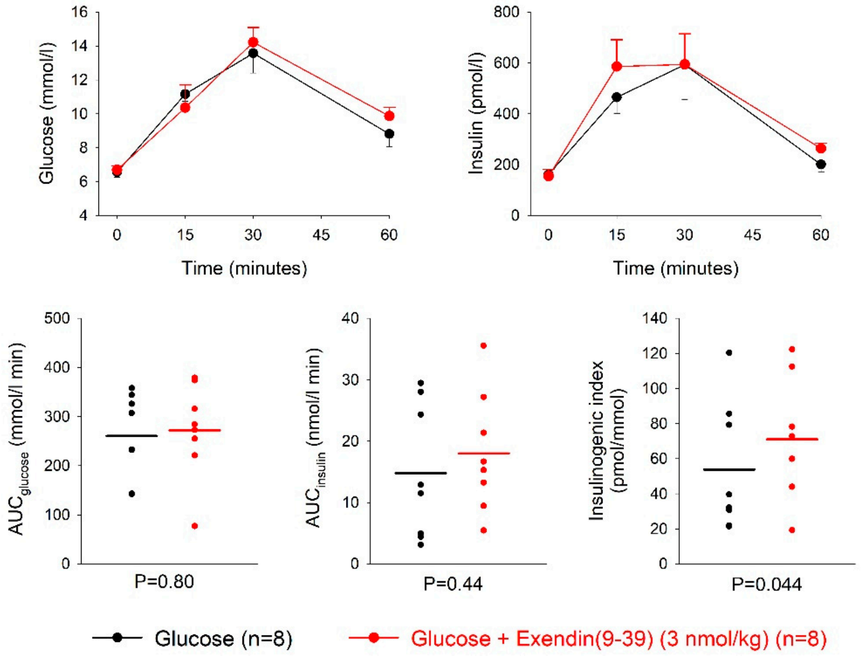

Supplementary Figure S3. Glucose and insulin levels, and individual data points for $AUC_{glucose}$, $AUC_{insulin}$ and insulinogenic index after intravenous administration of the GLP-1 receptor antagonist exendin(9-39) (3 nmol/kg) or saline four minutes after oral administration of 50mg glucose in normal mice. Means±SEM are shown. n indicates number of animals.
